# Supplementary figures and images for: The 2014 coral bleaching and freshwater flood events in Kāneʻohe Bay, Hawaiʻi
Source: PeerJ. 2015 Aug 4;3:e1136. doi: 10.7717/peerj.1136 (PMC4540025; doi:10.7717/peerj.1136)

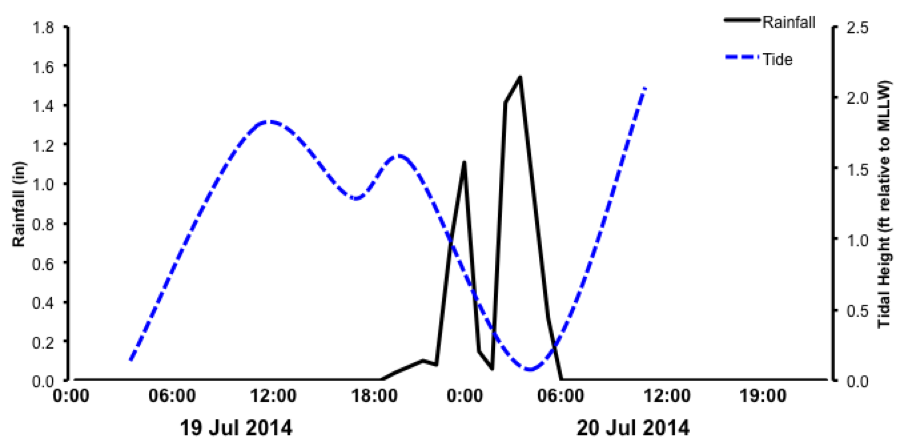

Supplement: Figure S1 — Hourly rainfall (black) data from the Wai‘āhole rain gauge and tide level (blue) in Kāneʻohe Bay on the night of July 19th into the early morning of July 20th. The largest amount of rainfall (1.6 inches in an hour) occurred during the lowest tide. [file peerj-03-1136-s002.png]

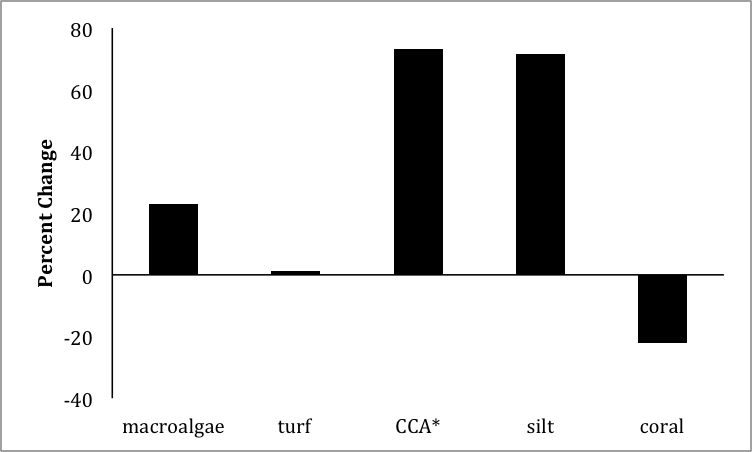

Supplement: Figure S2 — Mean (n = 5) percent change in major substrate types from quantitative surveys conducted between August 2014 and February 2015 on patch reef 37 in Kāneʻohe Bay, Hawaiʻi. ∗ CCA, calcareous coralline algae. [file peerj-03-1136-s003.png]

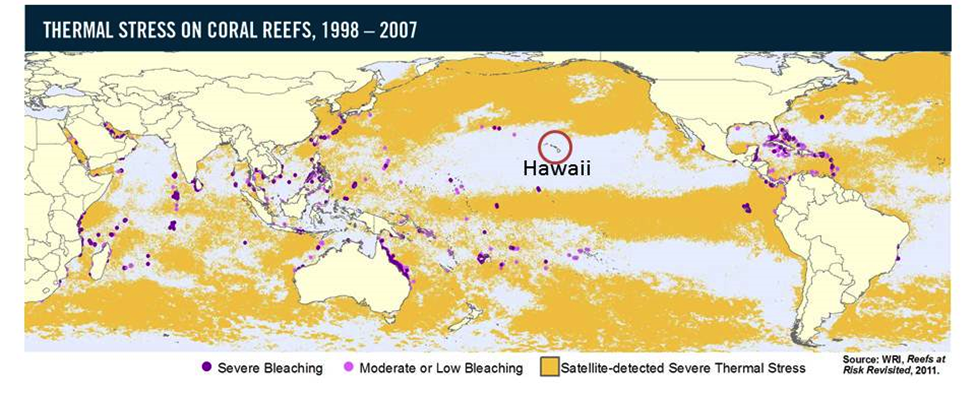

Supplement: Figure S3 — Hawaiian reefs largely escaped major bleaching events until recently (source: www.wri.org). Note that Hawaiʻi is located in a “sweet spot” in the central Pacific surrounded by deep ocean water away from continental shelves and effects of El Niño southern oscillation. [file peerj-03-1136-s004.png]
